# A qualitative assessment of business perspectives and tactics of tobacco and vape shop retailers in three communities in Orange County, CA, 2015–2016

**DOI:** 10.1186/s13690-018-0307-z

**Published:** 2018-10-18

**Authors:** Joshua S. Yang, Esther Lee

**Affiliations:** California State University, Fullerton, 800 N. State College Blvd, Fullerton, CA 92834 USA

**Keywords:** E-cigarettes, Vape shops, Marketing, Qualitative research

## Abstract

**Background:**

In response to the growing awareness and use of electronic nicotine delivery systems (ENDS), or e-cigarettes, the U.S. Food and Drug Administration asserted its regulatory authority over ENDS in May 2016. Federal, state, and local regulatory action on ENDS may have significant and unique impacts on specialty ENDS retailers, including tobacco and vape shops. The purpose of this study is to describe the commercial motivations of vape shops in minority communities as business entities whose financial interests and actions may be particularly impacted by regulation of ENDS.

**Methods:**

Specialty tobacco and vape retail stores in three minority communities were identified through an online search and community canvassing. Key informant interviews were conducted with tobacco and vape shop owners or managers discussing the business interests and tactics of selling ENDS for their store. Interview data were coded and analyzed for major themes.

**Results:**

Interviews with 18 tobacco shops and 9 vape shops were completed. Tobacco shops’ reasons for carrying e-cigarettes were business oriented, focused on maintaining their customer base. In comparison, vape shops opened because of the owner’s positive experiences with e-cigarettes and belief in the potential of e-cigarettes to help people quit or reduce smoking. Tobacco shops mainly see their customers as using e-cigarettes to quit smoking whereas vape shops reported their customers using e-cigarettes for more varied reasons. Tobacco shops are much more limited in their marketing than vape shops, which rely heavily on social media and experimentation with other forms of marketing.

**Conclusions:**

Tobacco shops and vape shops differ in their rationale and approaches to the business of e-cigarettes. Vape shops engage in a wide range of activities that stabilize their financial interest and increase their influence with customers and within the vape community. In order for regulatory policymaking and tobacco control interventions to maximize effectiveness, the actions of vape shops in promoting ENDS use and influencing policy debates must be taken into account.

## Background

Since their introduction in the United States in 2007, awareness and use of electronic nicotine delivery systems (ENDS), or e-cigarettes, among U.S. adults has been increasing. [1, 2] ENDS users may be exposed to several chemicals which have known adverse health effects including nicotine, carbonyl compounds, and volatile organic compounds and other heated and aerosolized ingredients, the health effects of which are not well understood [3]. Though the long-term health effects of ENDS use are unclear, recent studies have begun to better characterize the negative cardiovascular and pulmonary effects of ENDS use [4–7].

Rates of ENDS use among youth have been increasing as well, as they have become the most common form of tobacco used by U.S. middle school and high school students in 2014 [8], though recent data suggest vaping among teens may be beginning to decline [9]. In addition to potential health effects, ENDS use may also be a predictor for later combustible tobacco product use [10, 11], a pattern of particular concern for youth and young adults. Studies among adolescents suggest a higher odds of later initiation of combustible cigarette use for non-smoking ever e-cigarette users compared to non-smoking never e-cigarette users in early [12] and later [13] adolescence. Among adolescents and young adults ages 16–26 years old, another study found e-cigarette use was independently associated with progression to smoking after one year [14]. Among young adults 18–24 years of age, another study found that non-daily smokers who used e-cigarettes tend to smoke more cigarettes and do so more frequently [15]. A recent meta-analysis found a pooled odds ratio for subsequent cigarette smoking initiation was 3.62 (95% CI, 2.42–5.41) for ever e-cigarette users compared to never e-cigarette users [10].

In response to the growing use of an unregulated product and as part of its mandate under the Family Smoking Prevention and Tobacco Control Act, the U.S. Food and Drug Administration (FDA) issued a deeming rule asserting regulatory authority over ENDS in May 2016. FDA authority extends over “the manufacture, import, packaging, labeling, advertising, promotion, sale, and distribution of ENDS, including components and parts of ENDS but excluding accessories” [16]. Product manufacturers must submit tobacco health documents, ingredient lists, quantities of harmful and potentially harmful constituents, and include warning statements on packaging and advertisements. At the retail level, ENDS can only be sold to people 18 year old and older, cannot be given away as free samples, must be sold with a health warning statement on the package, and cannot be sold in vending machines unless they are in adult-only facilities. In addition, retail outlets can only display advertisements with a health warning statement and must check photo identification of everyone under age 27. Though an important foundation for tobacco efforts, the deeming rule left important regulatory gaps in place including exempting flavored nicotine products from regulation, placing no restriction on ENDS marketing, and delayed implementation of premarket review.

Though designed to reduce the negative impact of ENDS on population health, federal, state, and local regulatory action on ENDS also has significant impact on specialty ENDS retailers, also known as vape shops. The increase in ENDS use and projected growth in sales [17] has been accompanied by the emergence of vape shops, small businesses built on the sale of ENDS [18]. They differ from other outlets by providing a wider variety of products, selling newer generations of customizable devices, and allowing customers to sample ENDS products. Though the FDA assists small businesses in complying with new law, the regulatory requirements – especially for retailers who also manufacture their own products – may be considered by retailers a barrier to their business interests.

Studies focused on vape shops are limited. ENDS are widely available through different channels [19–22] and in-store marketing has been increasing [23, 24]. Previous studies show that vape shops play an important role in providing information [24, 25]. Vape shop owners and employees have positive attitudes towards and beliefs about ENDS [23–25] and engage in marketing which resembles tobacco industry marketing practices [26]. In addition to selling and providing information about ENDS, vape shops build rapport with customers and create an atmosphere around vaping which allows for interaction, builds a sense of community, and attracts customers [24, 27, 28].

Though reports suggest that the vape industry has responded to government efforts to assert regulatory authority [29], little empirical work has been conducted to identify the ways the vape industry or specific vape shops respond to or attempt to affect public health policy. Prior research on the tobacco industry [30] has shown that understanding the business interests, motivations, and tactics of corporate entities are critical to developing effective public health policies. This approach, however, is not limited to the tobacco industry [31]. Insight into the business interests and rationale of the vape industry and specific vape shops may lead to improved understanding of their response to increased regulation and thus lead to improved design, adoption, implementation, and enforcement of ENDS use prevention policies and programs. This is particularly true in ethnic minority communities which have higher rates of smoking than the general population [32] and for minority-owned small businesses which may present unique cultural and linguistic challenges for outreach. The purpose of this study is to describe the commercial motivations of vape shops in diverse communities as business entities whose financial interests may be particularly impacted by regulatory action on ENDS, contrasting them with tobacco shops that sell ENDS as a comparison group.

## Methods

Semi-structured interviews with managers or owners of tobacco or vape shops were conducted to identify key dimensions of their business. For the purposes of this study, “vape” shops are defined as retail outlets that sell ENDS and related products exclusively. Tobacco shops were defined as retail outlets for which conventional tobacco products (e.g., cigarettes, chewing tobacco) make up at least 50% of all products sold to delineate specialty tobacco shops from convenience or other retail outlets which sold tobacco. In order to be included in the study, tobacco shops also had to sell some type of ENDS.

### Cities

Three diverse, spatially contiguous cities were selected for inclusion in the study using purposive sampling [33] to address ENDS in racial minority communities important to tobacco control: Koreans, Vietnamese, and Mexicans. Koreans and Vietnamese smoke at higher rates than the general population and more than other Asian subgroups [32], and Latinos are the largest minority group in California. In Orange County, CA, the two largest racial/ethnic minority groups are Latinos (34.2%) and Asians (18.9%), with Mexicans the largest Latino subgroup in the county (29.3% of Orange County population; 85.8% of Latinos in Orange County), and Vietnamese (6.3% of Orange County population; 33.1% of Asians in Orange County) and Koreans (3.0% of Orange County population; 15.8% of Asians in Orange County) two of the largest Asian ethnic groups in the county [34]. The cities included in the sample were chosen based on their historical and documented enclaves of Korean (Garden Grove; “City A”), Vietnamese (Westminster; “City B”), and Mexican (Santa Ana; “City C”) populations [35, 36] and the substantially larger proportion of minority-owned businesses serving them than in the general population.

Based on 2015 U.S. Census estimates [34], 39.0% of the population in City A (174,721 total) was Asian, with 29.9% of the population identifying as Vietnamese and 2.8% as Korean. In City A, 36.7% of the population was Latino and 21% non-Hispanic White. Home to a two-mile stretch of Korean-owned businesses which attract Korean customers from the surrounding area [35], 52% of businesses in City A were Asian-owned in 2012. In City B, 48.2% of the population (91,719 total) was Asian, with 40.3% identifying as Vietnamese; 23.2% were Latino and 24.5% non-Hispanic White. A Vietnamese commercial district is centrally located in City B, but spreads to surrounding communities [35]. Of all businesses in City B, 54.2% were Asian-owned in 2012. In City C, 78.2% of the population (333,268 total) was Latino, with 72.6% identifying as Mexican; 10.6% were Asian and 9.2% non-Hispanic White. In City C, 31.8% of businesses were Hispanic-owned and 18.2% were Asian-owned in 2012. Thus, City A can be said to represent large Vietnamese and Korean populations; City B, a large Vietnamese population; and City C to represent a large Hispanic/Latino population.

### Sampling frame

To be included in this study, a retail outlet had to be either a vape or tobacco shop as defined above, and located within one of the three study cities. Internet searches using the terms “vape shop,” “tobacco shop,” and “e-cigarettes” were conducted using Yelp and Google Maps websites, a method similar to what was used in a previous study [37]. A total of 50 tobacco shops and 28 vape shops were identified. A community windshield survey [38] and subsequent field data collection found that half of all vape shops listed online had gone out of business. A random sample of 25 tobacco shops and all vape shops were selected for inclusion in the study. During data collection, four tobacco shops were out of business and were replaced in the sample. The final sample consisted of 25 tobacco shops (64%) and 14 vape shops (36%). Seven tobacco shops (4 in City A, 1 in City B, and 2 in City C) and 5 vape shops (1 in City A, 2 in City B, 2 in City C) refused to participate. Interviews were completed with owners or managers of 18 tobacco shops (10 in City A, 4 in City B, and 4 in City C) and 9 vape shops (6 in City A, 2 in City B, 1 in City C).

### Data collection procedure

Interviews were conducted with owners or managers in retail shops using a semi-structured interview guide. No tobacco shop owners agreed to be recorded and two vape shop owners refused to be recorded. Thus, all verbatim quotes below are from vape shop owners. For interviews which were not audio recorded, field notes were dictated into an audio recorder immediately after the interview. Interviews ranged from 5 to 45 min. Mean interview time of the seven audio recorded interviews with vape shop owners was 27 min. The research protocol was approved by the California State University Fullerton Institutional Review Board (HSR 15_0072, February 8, 2015). Interviews were conducted from August 2015 to May 2016.

Audio recordings of interviews and dictation of field notes were professionally transcribed, checked for accuracy by a research assistant, and imported into Atlas.ti 7 qualitative data analysis software for analysis [39]. Two research assistants used a preliminary coding scheme developed from the semi-structured interview guide to independently code the data. After an initial coding of a subset of interview transcripts, the research assistants and first author discussed coding results, clarified the meaning of codes, and refined codes. After a second round of coding to establish interrater reliability, Cohen’s κ was calculated to determine agreement between coders (κ = .771, *p* < 0.001). Research assistants then coded the entire data set with the final code list. Coded data were analyzed by the authors to identify patterns within themes established by codes. As patterns were identified, the number of mentions of a statement related to each pattern was counted across interviews.

## Results

Tobacco shops and vape shops differ in their rationale and approaches to the business of e-cigarettes (see Table 1). Tobacco shops’ reasons for carrying e-cigarettes were business oriented, focused on maintaining their customer base. In comparison, vape shops opened because of the owner’s positive experiences with e-cigarettes and belief in the potential of e-cigarettes to help people quit or reduce smoking. According to tobacco shop respondents, their customers use e-cigarettes primarily to quit smoking; vape shops reported their customers using e-cigarettes for a wider variety of reasons. Tobacco shops are much more limited in their marketing than vape shops, which rely on heavily on social media and experiment with other forms of marketing.

**Table 1:** Perceptions and strategies of tobacco and vape retail stores in Garden Grove, Santa Ana, and Westminster, CA, 2015–2016

	Tobacco shops (*n* = 18)	Vape shops (*n* = 9)
Why retailer carries ENDS^a^ products (tobacco shops) or opened an ENDS store		
Positive experience with or perceptions of ENDS	–	89%
Potential to help smokers quit or smoke less	–	44%
Important to carry them	39%	
Keep up with competitors	22%	
Not lose out on sales when customers ask for them	22%	
Follow a market trends	17%	
Stock a complete product line	17%	
Wholesaler recommended products	11%	
Perception of customer reasons for ENDS use		
To quit smoking	61%	78%
Reduce harm from smoking	–	44%
Socializing	–	56%
Flavor	–	33%
Watch weight	–	22%
Vape tricks	–	22%
Try something new	–	22%
Hobby	–	22%
Customizability of ENDS	–	11%
Convenience	6%	
Marketing activities used by retailer		
Social media	44%	100%
Do not market ENDS	56%	–
Word of mouth	22%	44%
Print media	–	33%
Radio	–	11%
Strategies employed by retailer to gain competitive advantage		
Customer service	11%	56%
Newness of products	–	44%
Price	–	33%
Quality of products	–	33%
Customizability	–	11%

^a^ ENDS – Electronic nicotine delivery system

### Bringing e-cigarettes to market

When asked why they started carrying e-cigarettes, the most common answer among tobacco shop owners was that customers began asking for them, so they felt it important to carry them. In addition to customer requests, tobacco shops also suggested that they needed to carry e-cigarettes in order to keep up with competitors, not to lose out on sales when customers came asking for them, follow a market trend, stock a complete product line, and because their wholesaler recommended they carry them.

Vape shops had more focused reasons for starting their shops. A common precursor to vape shops owners going into business was a positive experience with or perceptions of e-cigarettes and their potential to help quit or reduce smoking the number of cigarettes they smoked:

“[the owner said] 'I need vaping in order to actually cut down or quit cigarettes' and at the same time, he’s a businessman, so then he’s like, 'All right, let’s open up a vape shop.'” ---Key Informant 5.

“Before [the owner] opened the vape shop… his friends had him quit cigarettes, because he was a previous smoker. So once he quit cigarettes he then realized, oh wow, the products actually do work, so… when he was a hundred percent behind it then that’s when he opened the vape shop.” ---Key Informant 9.

Many also related the business opportunity to also helping people quit smoking or to smoke less:

“I was actually looking for an alternative for my father, who’s a heavy smoker… So I started looking deeper into the business aspect of it and got with two of my old business partners.”---Key Informant 6.

### Customers

When asked about their customers, the most common reason tobacco shops stated their customers used ENDS was to quit smoking, with only one respondent giving a separate reason for customer use of ENDS. Vape shops stated more reasons for e-cigarette use among their customers. The most commonly stated reason for use was to quit or reduce smoking:

“But the guys that come here, they’re not like the ladies. They’re not watching calories; they just want something to substitute cigarettes or maybe they’re doing a hybrid, like I said. They’re still kind of a smoker but they kind of reduce that smoking from maybe a pack to a day to maybe a couple cigarettes a day and they vape during the period of time.” ---Key Informant 1.

“We actually get a lot of people that come from other addictive things. They don’t want to do that stuff, they want to focus on something that’s clean, yeah. It’s not going to kill them, but, yeah, most of them are, are ex-smokers… Most of them are actually smokers trying to kick – to quit.” ---Key Informant 6.

“Most of them were ex-smokers. Some of them were people who normally would have turned to traditional cigarettes as a way of stress relief but found this as a safer option.” ---Key Informant 7.

There is also, however, a strong social component to ENDS use:

“…[customers] vape socially. It’s kind of like having a cigar every once in a while with your buddies while they’re having a drink or just chilling out, or people who go to hookah bars. They don’t smoke, but they like to do hookah. They don’t need it all the time, but they just want to have one just because it’s kind of trendy. And we get those too.” ---Key Informant 6.

“So the thing about vapers is that, if they see another vaper on the street, they are immediately friends – that’s just how it works – and they will talk about everything about vaping – what shops they go to, what flavors they vape, what they’re using – and usually that’s how you find out about new shops and where to get new stuff.” ---Key Informant 7.

“Girls… like one out of five girls that we get that are pretty much gung ho about vaping, other than that, they’re pretty much like tagalongs with their boyfriends.” ---Key Informant 9.

In addition, vape shops owners/managers suggested customers used e-cigarettes for the flavor, to watch their weight, to do tricks, to try something new, as a hobby, and because of the customizability of ENDS.

“[Girls] … if they’re watching their weight, they would rather vape e-liquid, which is zero milligrams and doesn’t have any nicotine, to substitute for eating that ice cream because you kind of get that same taste, but you don’t have all the calories…The guys are more the hobbyists. They want to have the most amount of clouds or the most amount of smoke produced and they’re more the high-end, the hobbyist kind of people that want to get the best out there.” ---Key Informant 1.

“People my age, they like, they like to see, or they like to have that hard hitting mechanical mod or hard hitting variable voltage mod, something that looks cool as well and then something that blows like a big cloud and has a good hit.” ---Key Informant 9.

### Marketing

Marketing of e-cigarettes by tobacco shops was limited, with most responding that they do not market e-cigarettes. Almost half have used social media, with others relying on word of mouth and price promotions. Vape shops utilized more diverse marketing channels, including radio, print media, and word of mouth. The most common form of marketing by vape shops is social media, which all vape shops cited using for marketing.

“We’ve done mailers, we’ve done radio, we’ve done everything you can think of...” ---Key Informant 6.

“Most of my customers just mouth to mouth, just sharing, 'Oh, yeah, I’ve been to this vape shop, and they’re just starting out, and so far, they look so good,' so that’s why I’m getting a lot of customers through that.” ---Key Informant 2.

“Most of our marketing is based off of social media; Facebook, Instagram, Twitter and whatnot, and then street signs, and then an occasional word of mouth, and that’s pretty much it.” ---Key Informant 8.

“I think social media is one of the big things. I have tried print. It didn’t give me that big of a response like social media. So I kind of decided to stick with social media… I have an Instagram page that I promote new stuff. As new stuff comes in, new juice comes in, I snap a couple pictures, put it up there for my followers… When you are vaping, you want to try the best new flavor out there.” ---Key Informant 1.

Promotions varied by type, including price promotions, loyalty programs, bundles, giveaways, and endorsements by “vape celebrities”:

“So what you do is you package it up into a bundle, you offer them a couple bucks off each one and you sell it as the whole shebang rather than just selling individual parts.” ---Key Informant 1.

“A lot of it is giveaways.” ---Key Informant 5.

“We did the breast cancer – it was a bike ride for breast cancer and we donated the most money so they give us that big trophy.” ---Key Informant 6.

“Just give as much free stuff as you can to people who are higher up in the community because, apparently, their word means everything. Because there’s, say, 10 or so higher ups in Orange County that people listen to non-stop… It’s a vaping celebrity.” ---Key Informant 7.

### Competitive advantage

Customer service and carrying new products are the two most common tactics which vape shops cited as helping them stay competitive in the retail space, compared to only 2 tobacco shops who cited any reason at all (both were customer service).

“[Customer service is] what tops everything else because, if somebody finds a shop that notices them as a person, remembers their name, treats them like an actual person, becomes their friend, that’s how you build the customer base in this industry. Because you don’t want someone who just gets their stuff and leaves. You want to be able to hang out with the customer in the shop and talk to them for a while – things like that.” ---Key Informant 7.

“It’s the latest, hottest products. It’s the new stuff that comes out that always gets pushed out. It’s crazy, every month there’s new products, every month, never ending…And you’re looking for the latest and greatest – just vaporizers, something that’s going to be easy to use and reliable and quality, and things just getting better and better, more advanced, more flavor, less hassle, less maintenance, and stuff like that.” ---Key Informant 6.

In addition, vape shops compete to provide the best products at the best price, focusing on quality, value, and customizability:

“There are so many other vape shops around here and some of them now, with so much saturation, most of them sell the exact same products. So it really comes down to customer service, prices, and the feel.” ---Key Informant 1.

## Discussion

Tobacco and vape shop owners and managers both discussed ENDS in financial terms, but the nature of their investment was different. Tobacco shops rely on conventional tobacco products with ENDS as an ancillary product. Vape shops, on the other hand, place a larger investment in ENDS. In addition to the income generating role of their shops, vape shop owners and managers articulated a social purpose to their stores: to help people quit smoking by providing the best products and knowledgeable, customized guidance on the many products in the ENDS market. This social function is reinforced through experiences of successfully quitting or reducing the number of cigarettes smoked by themselves or someone they know, and seeing customers successfully eliminate or reduce tobacco use. The effect of FDA regulation could therefore be expected to have a more significant impact on vape shops than tobacco shops, especially those which develop their own products. Thus, the vape industry may be more outspoken about FDA regulation, and its specific positions influenced by the high level of financial investment in ENDS and a feeling among the vape community that their product does a social good by helping people quit smoking. In addition, study respondents characterized their products in contrast to conventional tobacco products and ENDS sold by large tobacco companies; thus, regulatory efforts which group the tobacco industry and the small business component of the vape industry may be resisted in part because of wanting to maintain a distinction from the tobacco industry.

A key approach in the FDA deeming rule is to protect young people from potential dangers of ENDS use. Yet the FDA has not proposed rules to regulate the use of social media – a platform popular among youth [40, 41] – to market ENDS, which the present study shows is the marketing channel of choice among vape shops. Though vape shops may reject the idea that they advertise and market to youth, easy access to vape shops’ social media accounts may nonetheless impact youth attitudes and behaviors. In addition, our study suggests that vape shops use a wide variety of marketing approaches which are not included in the current regulatory framework such as endorsements, street signs, and sponsorships. Minority youth in the study communities may be particularly impacted by vape shops’ widespread practice of posting “new and cool” products on social media and exposed to informal advertising outside of retail settings. Regulatory authorities will need to consider efforts to reduce the impact of social media and “below the line” marketing to protect youth and adolescents.

In order to gain a competitive advantage in the marketplace, vape shop owners and managers commonly stated that they distinguish their stores from others through customer service and new products. These findings support previous studies which found vape shops build rapport with customers and create an atmosphere around vaping which allows for interaction, builds a sense of community, and attracts customers [24, 27, 28]. The relational dimension of vape shops may contribute to the spread of information about ENDS including new products, forms of use, and health-related issues. Thus, while the FDA has required warning labels on products and advertisements, it cannot control what vape shop owners and staff communicate to their customers or where they get information. Vape shops are not only retail outlets where products are made available; they also act as community foci for information about ENDS. The FDA and other regulatory bodies should not only enact and enforce regulation over ENDS but also establish communication channels to update regulated entities about new information and evidence about the behavioral and health effects of ENDS. This needs to be done with an appreciation for the diversity of retailers and the communities in which they are embedded. Local health agencies and community health organizations may be best suited to understand the needs of diverse communities and develop culturally competent materials for vape shops in minority communities and their customers.

The anecdotal experiences of seeing people who use ENDS to quit or reduce smoking in their own lives or among customers reinforces vape shop owner and managers’ perception that e-cigarettes work as cessation devices. Yet, they also report a wide range of reasons for which customers use ENDS including flavor, tricks, as a hobby, and to watch one’s weight. The appeal of flavored ENDS products suggests that flavoring may be a reason for initiating or continuing ENDS use, and thus the FDA should consider asserting regulatory authority of flavored ENDS products, especially with the particular appeal of flavorings to youth. ENDS are also appealing for their novelty and customizability. Thus, with limited evidence on whether an unregulated ENDS market leads to improved population health [42–44], it is important but not sufficient to develop and implement educational efforts on the effectiveness of ENDS as cessation devices and their potential as gateway products to combustible tobacco use. Interventions must denormalize ENDS across racial/ethnic groups, targeting the social basis of ENDS use and non-health related reasons people use ENDS.

With new regulations, it will be important to monitor how ENDS retailers adapt to changing regulatory environments. Though vape shops report seeking competitive advantage through traditional means, reliance on social media shows that vape shops also utilize new technologies and methods to reach customers. Monitoring novel and emerging business tactics of the vape industry and unintended policy effects will be needed.

One limitation of this study is reliance on self-report of store owners and managers. Future studies should focus directly on the attitudes and behaviors of customers. Also, more than one-third of all vape shops refused to participate; their responses may have differed from owners or managers who did participate. Non-response may have been influenced by the relatively large immigrant population in each city and varying levels of acculturation of owners/managers in enclave communities. These factors may have reduced their comfort with participating in a research study conducted by a community outsider. The study was limited in its geographical scope and number of vape shops included in the study, affecting the generalizability of the results to other areas. Another limitation to generalizability is the focus on three minority communities. Future surveys should include retail outlets for tobacco products to generalize prevalence of business strategies across a wider group of tobacco and vape retail outlets, measure their effect on customers, and examine the degree to which different types of retail outlets appeal to distinct market segments.

## Conclusion

Limited evidence on the overall population health effects of e-cigarettes has led to a highly contested regulatory space for ENDS. Due to their unique financial and social interest, vape shops may play a significant role in resisting efforts to regulate ENDS and acting as a source of ENDS-related health information for customers, more so than tobacco shops because of the singular focus on ENDS. Vape shops engage in a wide range of activities that stabilize their financial interest and increase their influence with customers and within the vape community. In order for regulatory policymaking and tobacco control interventions to maximize effectiveness, the actions of vape shops in promoting ENDS use and influencing policy debates must be taken into account.

## Abbreviations

ENDS: Electronic nicotine delivery system
FDA: U.S. Food and Drug Administration
